# Glycemic index of a nutritional supplement designed for people with chronic kidney disease

**DOI:** 10.1002/fsn3.3495

**Published:** 2023-08-03

**Authors:** Bhoite Rachana, Shanmugam Shobana, Pratti Varalakshmi Lalithya, Vasudevan Sudha, Satyavrat Vinita, Rajagopal Gayathri, Natarajan Kalpana, Mohan Anjana Ranjit, Mohan Viswanathan

**Affiliations:** ^1^ Dr. Reddy's Laboratories Pvt Ltd. Hyderabad Telangana India; ^2^ Department of Foods Nutrition & Dietetics Research, Madras Diabetes Research Foundation Chennai Tamil Nadu India; ^3^ Department of Diabetology Dr. Mohan's Diabetes Specialities Centre Chennai Tamil Nadu India

**Keywords:** chronic kidney disease, glycemic index, nephropathy, nutritional supplement, renal nutrition

## Abstract

The study was carried out to measure the glycemic index (GI) of an oral food supplement for people with CKD as well as on patients on maintenance dialysis. The study was conducted as per international protocols for testing GI, was approved by the local institutional ethics committee, and was registered with the Clinical Trial Registry of India (CTRI). This was a crossover randomized controlled study which enrolled 15 participants between the ages of 18 and 45 years. The participants were randomly allotted to one group that consumed either the reference food (27.5 g of glucose monohydrate) or 118 g of the nutritional supplement which contained 25 g of available carbohydrates. Fasting capillary blood samples as well as blood samples at different time intervals as per the GI protocol, after consumption of either the supplement or the reference food were taken from the participants. Each testing day was separated by a 3‐day washout period. GI was calculated from the incremental area under the blood glucose response elicited by the nutritional supplement as a percentage of the response after the consumption of 25 g of glucose (27.5 g of glucose monohydrate) by the same participant using a standard formula. The GI of the nutritional supplement was calculated to be 10.3 ± 2.0 which is considered to be low as per international GI testing standards. The product was created to supplement the diet of people with CKD at different stages and to help prevent the progression from CKD to ESRD as well as the risk for CVD. This product was found to have a low GI which is desirable for people with CKD as well as diabetics in general who are at risk for developing CKD.

## Graphical abstract

A study was carried out to measure the glycemic index (GI) of an oral food supplement for people with chronic kidney disease (CKD) as well as on patients on maintenance dialysis. The GI of the nutritional supplement was calculated to be 10.3 ± 2.0 which is considered to be low as per international GI testing standards.

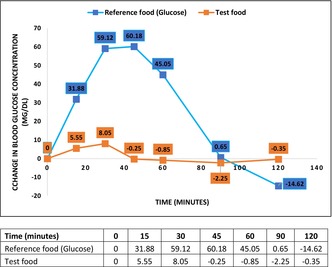

## 1 INTRODUCTION

Chronic kidney disease (CKD) is a condition where there is decreased kidney function which is usually caused by diabetes and hypertension (Kramer, 2019; Webster et al., 2017) (United States Renal Data System, 2022). The prevalence of CKD across the world was 9.1% in 2017, i.e., around 700 million people with the disease (Bikbov et al., 2020).

With the rise in the prevalence of diabetes in pandemic proportions across the world, there has been a parallel rise in the prevalence of diabetic nephropathy. Globally diabetic nephropathy is a leading cause of end‐stage renal disease (ESRD) (Benjamin & Lappin, 2021) and approximately 40% of people with type 2 diabetes mellitus (DM2) end up developing diabetic nephropathy (Reutens, 2013) (Hussain et al., 2019).

High blood glucose level is not only an independent risk factor for the development of diabetic nephropathy but the most important one (Caramori et al., 2013). There are well designed randomized controlled studies that have shown the effect of blood glucose control on delayed onset of markers of CKD like albuminuria in DM2 (Ismail‐Beigi et al., 2010) (Duckworth et al., 2009).

CKD is a silent disease and studies have shown that more than 90% of people suffering from stage 2 or 3 CKD were not aware of their condition till they were late into the disease (Szczech et al., 2014) (Bakris et al., 2019). Another survey done on people with diabetes, who were detected to have stage 3 of 4 of CKD found that only 15% of them were actually aware that they have any kidney disease (Dharmarajan et al., 2017). Studies have shown that increased consumption of drinks high in sugar was associated with an increased risk for DM2 and obesity which have been linked with the progression toward ESRD in patients with CKD (Schulze et al., 2004), two major risk factors for progression toward chronic kidney disease (Fox et al., 2004). Consumption of sugary drinks has been shown to be associated with chronic kidney disease in well‐conducted studies (Saldana et al., 2007) (Shoham et al., 2008). With these established facts that DM2 has a direct correlation with CKD as well as its progression to ESRD, any intervention that helps in reducing the risk of DM2 and its complication can be logically thought to help in the incidence of ESRD too. A large randomized controlled trial looked at the impact of a multifactorial as well as a multidisciplinary team approach in patients with stages 3 and 4 CKD (Fogelfeld et al., 2017). An integrative approach comprising intensive diabetes and renal care in combination with behavioral as well as dietary interventions was used to manage the patients. The study showed that the patients with stages 3 and 4 CKD who were part of the intervention group and received the integrated intensive management had increased chances of achieving an HbA1C below 7% with a greater decrease in the albumin–creatinine ratio. The study further showed that only 13% of the patients in the intervention group progressed to ESRD in comparison with 28% in the control group, which roughly accounted for more than a 50% reduction in the chances of progressing toward ESRD in the intervention group. The study demonstrated that besides pharmacological interventions, there is a big role that dietary interventions could play especially in reducing hyperglycemia, albumin–creatinine ratio, and thus progression of CKD to ESRD.

A systematic review estimated that more than 24% of cases with CKD in industrialized countries could be attributed to nutritional factors which are a part of modifiable risk factors for the development of both CKD and its progression to ESRD (Wang et al., 2008). A 3‐fold increase in the incidence of CKD over 5 years was seen in a group of people consuming energy‐dense sources of carbohydrates which underlies the need for assessing the quality of carbohydrates, especially in people who are at risk of developing CKD or progressing toward ESRD (Gopinath et al., 2011). Although ESRD is a logical progression from CKD in people with uncontrolled diabetes mellitus, it has been seen that CKD is also associated with cardiovascular risk factors and majority of patients with CKD will not reach ESRD as they may die of cardiovascular risk factors that may precede it (USRDS, 2018). Therefore, interventions to prevent cardiovascular risk factors or to manage the same should be given a major impetus while designing the management for people with CKD. Foods containing different types of carbohydrates can have variable glycemic responses, where some foods can cause sudden surge as well as fall in blood glucose levels postconsumption and some foods can cause slow rise as well as fall in blood glucose levels. The quality of carbohydrates can be assessed by measuring the dietary glycemic index (GI) which represents the rate of rise in blood glucose levels after consumption of carbohydrates (Gopinath et al., 2011; Jenkins et al., 1987).

People with CKD whose diets are deficient in protein and energy requirements are often recommended oral nutritional supplements (Jensen, 2013). It was imperative that any oral nutritional supplement have a low GI besides fulfilling the protein as well as micronutrient requirements for this particular group of people. The present study, thus, aimed at analyzing the quality of carbohydrates in a healthy food drink tailored for people with CKD. This study measured the GI of the product for it to be a suitable nutritional supplement for people with CKD as well as for people with DM2, who are at a higher risk for developing CKD.

## 2 MATERIALS AND METHODS

### 2.1 GI testing

This study was conducted as per protocols recognized by Food and Agricultural Organization/World Health Organization (FAO/WHO, 1998), as per guidelines by international dietary carbohydrate task force for the measurement of GI in carbohydrate‐rich foods for GI methods (Brouns et al., 2005), as well as methods recognized by International Organization for Standardization (ISO) (ISO, 2010) (Henry et al., 2008). This was a crossover randomized controlled study as per international guidelines for GI studies and the participants from the study were recruited from a roster of the GI testing center of Madras Diabetes Research Foundation (MDRF) which was the site where the study was conducted. Participants were free‐living individuals from the community between 18 and 45 years of age. The inclusion criteria were males or females *with a BMI of less than or equal to 22.9* kg/ m^2^. People with any known food allergy or intolerance, with a history of any medication known to have an effect on glucose metabolism, with any specific dietary restrictions, with a known history of diabetes mellitus, with a history of any disease or drug intake that influenced digestions as well as absorption of nutrients, with a history of any medical or surgical event in the last 3 months, and mothers who were pregnant or lactating, were excluded from the study. *The study was approved by the institutional ethics committee of MDRF, Chennai, India* and was conducted as per the good clinical practices and as per the declaration of Helsinki. *Informed consent was taken from each participant before enrolling them in the study*. The study was registered in the Clinical Trial Registry of India (CTRI); *CTRI/2021/08/035929* (CTRI, n.d.), *which is linked to the WHO clinical trial registry platform* (WHO, n.d.).

### 2.2 Sample size

As per recommendations for the estimation of GI, a sample size of 10 participants was good enough (Brouns et al., 2005). This study enrolled 15 participants anticipating dropouts if any.

### 2.3 Intervention

As routine procedure participants were familiarized with capillary blood sampling prior to the start of the study. The study included a total of 15 participants who were randomly allocated to either group A or B. The reference food containing 25 g of available carbohydrates (27.5 g of glucose monohydrate) was given on three occasions (beginning‐R1, middle‐R2, and at the end of the study‐R3). The test food feeding (containing 25 g of available carbohydrates containing nutrition supplement powder) to both groups was randomized in such a way that group A consumed test food between R1 and R2, while group B participants consumed between R2 and R3. All the participants were hence had four visit days. Each visit day was separated by three washout days to prevent the carry‐over effect of the visit day's reference or test food intake. A 24‐h dietary recall was done, and the dietary intakes were assessed by trained research assistants with the help of visual food atlas with real food images, portion tools, and sizes to assist in the accuracy of the dietary intake assessment (Sudha et al., 2006). A history of physical activity was taken for the day prior to the testing using a standardized physical activity questionnaire (Anjana et al., 2015). A brief history of, smoking and alcohol were obtained for the pretest dates, and it was ensured that the participants refrained from smoking and alcohol during the study period. Participants were asked to report to the GI testing center each test day in the morning after a 10‐ to 12‐h overnight fast, to take the scheduled reference food or test food.

### 2.4 Estimation of GI

The operational definition for GI for this study is the incremental area under the blood glucose response elicited by 25 or 50 g of available carbohydrate‐containing test food portion expressed as a percentage of the response after the consumption of 25 or 50 g of anhydrous glucose by the same participant (Wolever, 2006) and is the comparison of mass to mass of carbohydrates in single foods.

The test product was a protein‐rich, proprietary formulation which is targeted for people with CKD and manufactured by Dr. Reddy's laboratories limited, India, the nutrient composition of which is given in Table 1. Proximate composition, dietary fiber, and available carbohydrate content (direct measurement) of the test product were estimated at the Food Quality Analysis Lab in MDRF. The test food quantity of 118 g was needed to obtain 25 g of available carbohydrates based on the analytical values. A 27.5 g of glucose dissolved in 125 mL of water was given as the reference test food to the participants and 118 g of the test food mixed with 531 mL of water was given as the test food. Participants were given an additional 125‐mL water with the test portion.

**TABLE 1:** Nutritional composition of the nutritional supplement.

Nutrients	Unit	100 g
Energy	kcal	440
Protein^a^	g	48
Carbohydrate	g	20.3
Total sugars	g	0
Added sugar (sucrose)	g	0
Total fat	g	17.2
Monounsaturated fatty acids	g	4
Polyunsaturated fatty acids	g	0.9
Saturated fatty acids	g	9.7
Cholesterol	mg	<1.0
Trans fatty acids	g	<0.1
Dietary fiber	g	6
Sodium	mg	251
Vitamins		
Vitamin C	mg	78
Vitamin B5	mg	5.5
Vitamin E	mg	11.2
Vitamin B6	mg	1.5
Vitamin B2	mg	1.55
Vitamin B1	mg	1.55
Vitamin B3	mg	11.5
Vitamin A	mcg	253
Folic acid	mcg	174
Vitamin K	mcg	23
Biotin	mcg	26
Vitamin D	mcg	8
Vitamin B12	mcg	2.2
Minerals		
Chloride	mg	160
Magnesium	mg	56
Can describe Iron	mg	5.05
Zinc	mg	2.5
Manganese	mg	1.8
Selenium	mcg	38
Calcium	mg	282
Chromium	mcg	57
Phosphorous	mg	213
Copper	mcg	562
Potassium	mg	445.5
Other nutrients		
Choline	mg	169
L‐Carnitine	mg	70
L‐Taurine	mg	40

^a^ Whey protein isolate contributed to 80% and soy protein isolate to 20% of total proteins.

Fasting blood samples were taken at −5 min and 0 min by finger‐prick, using an automatic lancet device before consumption of the food and the baseline value taken was a mean of these two values. The participants then consumed 25 g available carbohydrate portion of the reference or the test food portion as per the schedule and randomization. The first bite/sip in the mouth was set as time 0 and the capillary blood samples were taken at 15, 30, 45, 60, 90, and 120 min.

GI was calculated using the following formula (Brouns et al., 2005) (ISO, 2010):

$$\mathrm{GI}\;\text{value of test food}\;\left(\%\right)=\frac{\text{Blood}\;\text{glucose IAUC value for the test food}}{\text{IAUC}\;\text{value of the reference food}}\times 100$$

The Incremental Area under the Curve (IAUC) of blood glucose for the reference and test food were calculated geometrically using the trapezoid rule, ignoring the area below the fasting (FAO/WHO, 1998) (Brouns et al., 2005) (ISO, 2010) (Henry et al., 2008) (Augustin et al., 2015). The mean and standard errors (SEM) of the IAUC for the reference and test food were calculated. GI values were calculated by expressing each subject's IAUC after the test food as a percentage of the same subject's mean reference IAUC. The mean of the resulting value was the GI of the test food (Table 3).

## 3 RESULTS

**FIGURE 1:**
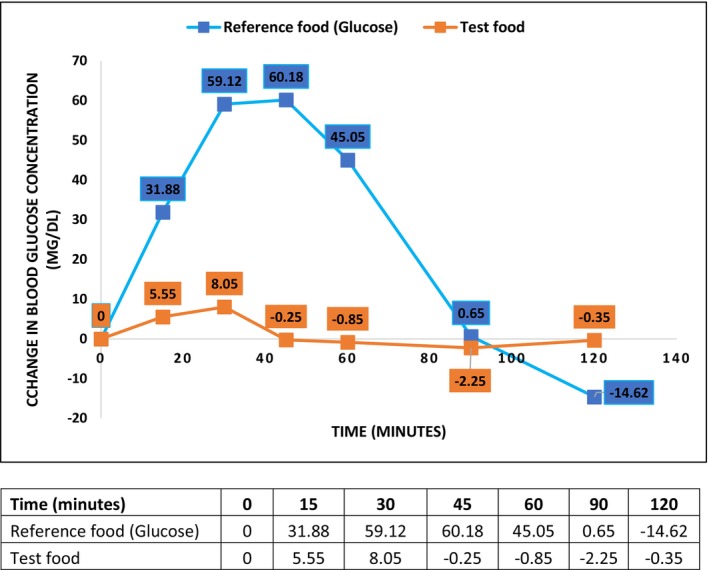
Change in blood glucose between reference food (glucose) and nutritional supplement (test food) over 2 h.

A total of 15 participants (eight females and seven males) were enrolled for this and out of which data from 10 were used for the final analysis (Figure 1). The mean age of the study participants was 27.5 years, and their mean BMI was 20.9 kg/m^2^. The baseline characteristics of the participants in the study are given in Table 2.

**TABLE 2:** Baseline characteristics of study participants.

Characteristics	Mean ± SD
Age (Years)	27.5 ± 4.5
BMI (kg/m^2^)	20.9 ± 1.2
Male (*n*%)	7 (46.7%)
Female (*n*%)	8 (53.3%)
Fasting Blood Sugar (mg/dL)	89

*Note*: *n* = No. of healthy volunteers who participated in the study.

Abbreviations: BMI, Body mass index; SD, Standard deviation.

Out of the 15 participants initially enrolled for the study, three participants dropped out from it and the data of one participant who could not consume the full test food was removed from the final analysis. As per standard methodology individual data of all participants, one participant with a coefficient of variation (CV) >30% (for reference glucose to reduce intraindividual variability) was removed as an outlier and did not find any individual values of GI > mean GI ± 2SD as outliers (Altman, 1991). The GI of the test product was calculated to be 10.3 ± 2.0 (Table 3) which is classified under the category of low GI foods (Vega‐López et al., 2018).

**TABLE 3:** Individual Mean IAUC of reference (Glucose) and GI of the Test food.

S. No	Age in years	BMI kg/m^2^	Mean IAUC – reference (mg/dL × min)	Glycemic index – test food (*n* = 10)
1	30	20.8	5119.1	16.9
2	38	20.9	3620.9	14.4
3	29	18.7	5227.4	15.3
4	30	21.6	3983.0	7.5
5	24	21.6	1719.0	7.9
6	28	22.0	2352.5	^b^
7	31	21.3	2367.0	20.8
8	26	22.5	3072.1	0.1
9	28	21.6	3015.2	6.1
10	32	20.1	3471.5	7.3
11	21	19.4	3719.4	^c^
12	25	18.6	3653.2	^a^
13	26	21.3	2623.7	^b^
14	22	22.1	3127.6	7.1
15	22	20.5	1274.3	^b^
Mean	27.5	20.9	3223.1	10.3
SEM	1.2	0.3	283.1	2.0

Abbreviations: SEM, Standard Error of Mean.

^a^ Did not consume completely.

^b^ Dropout.

^c^ Outliers >30% CV.

## 4 DISCUSSION

Glycemic index is the most reliable predictor of glycemic variations. Foods having GI ≥70 on the glucose scale are considered high‐GI foods, whereas those that have GI ≤55 on the glucose scale are considered low‐GI foods. It is important to highlight here that low‐GI foods provoke lesser blood glucose fluctuations than high‐GI foods over the day (Augustin et al., 2015).

Glycemic Index is considered to be a tool to help people with diabetes choose healthier carbohydrate food choices, was established more than 40 years back in 1981 (Jenkins et al., 1981), and has been used ever since by clinicians as well as nutritionists for the value it provides while prescribing a diet. The GI of the nutritional supplement (test food) used in the study was found to be low as per the GI classification (Augustin et al., 2015) and the GI value was 10.3 ± 2.0 (Table 3). The ingredients used to design the nutritional supplement, such as medium‐chain triglycerides (MCTs) and the combination of protein used could be the reason for low GI. This supplement is specially designed to meet the high protein requirement of CKD patients undergoing dialysis as per international guidelines (Cano et al., 2006) (Ikizler et al., 2020). One of the major challenges faced by physicians is glycemic control in people with CKD which comprises prescribing both medications and proper dietary advice. Dietary interventions should consist of not only correct combinations of protein and energy requirements for every stage of CKD but should also take into consideration the quality of both carbohydrates and proteins. A healthy diet may help in reducing the progression of CKD toward ESRD and combined with proper medications may go a long way in helping people with CKD (Stevens & Levin, 2013). A systematic review of cohort studies showed that diets with high GI and glycemic load (GL) were independently associated with increased risk of DM2 in healthy populations (Livesey et al., 2019). A seminal paper that examined evidence based on Bradford Hill criteria for causality (Hill, 1965) demonstrated GI and GL as causal factors for the risk of developing DM2 (Stevens & Levin, 2013). Alternatively, a cohort study showed that consumption of carbohydrates with low GI reduced the risk of developing CKD by 50% in the population studied (Gopinath et al., 2011).

Dietary interventions for people with CKD must be tailored to meet their protein and energy requirements while slowing the progression from CKD to ESRD. Interventions to prevent cardiovascular risk factors or to manage the same should have a major impetus while designing the management of people with CKD as most of the people with CKD die of cardiovascular risk factors rather than of ESRD. Genes play a big role in the incidence of ESRD in people under 50 years of age but it has also been seen that majority of cases with ESRD are associated with modifiable nutritional risk factors (Kramer, 2019). Management of cardiovascular disease risk factors such as hypertension, obesity, diabetes, and dyslipidemia should be an essential part for the management of people with CKD. A systematic review of randomized controlled trials showed that a low GI diet helped improved glycemic control in people with diabetes (Thomas & Elliott, 2009). A large multicontinental cohort study which analyzed data from 137,851 participants between 35 and 70 years of age from five continents showed that participants who consumed diets low in GI and GL had a significantly lower risk of cardiovascular disease and all‐cause mortality in comparison with participants who consumed diets high in GI and GL (Jenkins et al., 2021). A recently conducted systematic review also suggested that low GI foods can help improve risk factors for CVD like dyslipidemia, adiposity, blood pressure, and inflammation, even in people treated with medications for diabetes (Chiavaroli et al., 2021).

Indian diets are primarily low in both quality as well as quantity of protein and more than 70% of Indians have a protein‐deficient diet (Manish M., 2015). People with CKD and ESRD also suffer from protein malnutrition which is associated with increased morbidity and mortality. It is recommended that people with CKD not on dialysis have 0.6–0.8 g/kg body weight of protein per day and people on dialysis have 1–1.2 g/kg body weight of proteins per day, along with essential micronutrients (Zha & Qian, 2017). The International Society of Renal Nutrition and Metabolism (ISRNM) has coined “protein‐energy wasting” (PEW) for the loss of muscle as well as stored energy and “kidney wasting disease” for PEW in acute kidney disease (AKD) or CKD irrespective of the cause(s) (Fouque, Kalantar‐Zadeh et al., 2008). People suffering from CKD especially those who require dialysis tend to have malnutrition more so because this condition is usually accompanied by anorexia and that is why oral supplementation with macro‐ as well as micronutrients is recommended for this group of people. A cohort study done on patients with CKD requiring hemodialysis showed that more than 50% of the patients who were undernourished and were recommended medical nutritional therapy had better clinical outcomes (Tan et al., 2016). Among other biochemical and anthropometric measurements to determine PEW in AKD or CKD, dietary intake of proteins and carbohydrates form a part of the major criteria. Unintentional low dietary intake of protein lower than 0.8 g/kg body weight of proteins per day for at least 2 months for patients requiring dialysis, lower than 0.6 g/kg body weight of proteins per day for patients with stages of 2–5 CKD stages or unintentional low consumption of daily energy intake of less than 25 kcal/kg body weight per day for at least 2 months are important criteria to diagnose PEW in AKD or CKD (Fouque, McKenzie et al., 2008). Patients suffering from CKD, on either hemodialysis or peritoneal dialysis, have poor appetite mainly due to the presence of uremic toxins, increased catabolism, oxidative stress as well as the presence of other comorbidities like diabetes (Burrowes et al., 2003) (Malgorzewicz et al., 2016) and therefore may require specialized supplementation. Around 11% of patients who were started on specialized predialysis care were seen to suffer from moderate protein energy malnutrition (Westland et al., 2015). A systematic review of randomized controlled trials that analyzed the effect of oral protein‐based supplements in people with CKD requiring dialysis found that participants who were on protein‐rich food supplements improved nutritional markers as well as an increase in midarm muscle circumference which is one of the clinical measurements for PEW (Mah et al., 2020). Nutritional supplements specially designed for patients undergoing maintenance hemodialysis have been shown to prevent malnutrition, reduce hospitalization days, as well as improve quality of life (QOL) in these patients (Fouque, McKenzie et al., 2008) (Kalantar‐Zadeh et al., 2011) (Wilson et al., 2001). Whey or soy protein‐based supplements have helped improve health outcomes like, increased muscle mass to improve physical function and have reduced inflammatory markers like C‐reactive protein (CRP) and interleukin 6 (Il‐6) in patients with CKD on maintenance hemodialysis (Tomayko et al., 2015). The nutritional product in this study was a whey and soy protein‐based supplement, designed to support patients with CKD or undergoing dialysis, with other essential nutrients required on a daily basis, (Table 2). The product was created to supplement the diet of people with CKD at different stages and to help prevent the progression from CKD to ESRD as well as risk for CVD. A carbohydrate‐rich food is considered to have a low GI if the GI is <55 (Eleazu, 2016), the nutritional product studied in this study had a GI of 10.3 and therefore can be considered as a low GI food.

## 5 CONCLUSION

Consuming proper proportion of macronutrients (carbohydrates, proteins, and fats) is vital for sustaining caloric sufficiency, protein sparing, and achieving balance in nutrition. The test product is designed specifically to meet the nutritional needs of patients with CKD at different stages and can be used adjuvant to their daily diet to help fill the nutritional gap, especially their higher protein needs and to help prevent the progression from CKD to ESRD as well as risk for CVD. This product was found to have a low GI (10.3 ± 2.0) which is desirable for people with CKD as well as diabetics in general who are at risk for developing CKD.

## 6 PRACTICAL APPLICATION

The oral food supplement used in this study was a whey and soy‐based product with other essential nutrients, which was specially designed for people at different stages of CKD. The GI for the food supplement was assessed as per international norms for the testing of GI in carbohydrate‐rich foods. The study demonstrated that the oral nutritional supplement had a low GI as per international GI testing norms, which is desirable for people with CKD as well as diabetics in general who are at risk for developing CKD.

## AUTHOR CONTRIBUTIONS

**Bhoite Rachana:** Conceptualization (equal); supervision (equal); validation (equal); writing – review and editing (equal). **Shanmugam Shobana:** Conceptualization (equal); supervision (equal); validation (equal); visualization (equal). **Pratti Varalakshmi Lalithya:** Supervision (equal); validation (equal); writing – review and editing (equal). **Vasudevan Sudha:** Conceptualization (equal); supervision (equal); validation (equal); visualization (equal); writing – review and editing (equal). **Satyvarat Vinita:** Funding acquisition (equal); supervision (equal); writing – review and editing (equal). **Rajagopal Gayathri:** Data curation (equal); methodology (equal). **Natarajan Kalpana:** Data curation (equal); formal analysis (equal); methodology (equal); software (equal). **Mohan Anjana Ranjit:** Validation (equal); writing – review and editing (equal).

## FUNDING INFORMATION

This research was funded by Dr. Reddy's Laboratories Ltd.

## CONFLICT OF INTEREST STATEMENT

None to report.

## Data Availability

The data that support the findings of this study are available on request from the corresponding author. The data are not publicly available due to privacy or ethical restrictions.
